# Activation of the Complement System: Morphological Differences in the Surface of PIBCA Nanoparticles Coated with Polysaccharides

**DOI:** 10.1021/acsnanoscienceau.5c00196

**Published:** 2026-03-27

**Authors:** Iago Dillion Lima Cavalcanti, Vinícius Alexandre Fiaia Costa, Danilo Rosa Seixas, Bruno Junior Neves, Francisco Humberto Xavier-Junior, Mariane Cajubá de Britto Lira Nogueira, Nereide Stela Santos Magalhães, Eloisa Berbel Manaia, Gilles Ponchel

**Affiliations:** 1 Instituto Keizo-Asami (iLIKA), Universidade Federal de Pernambuco (UFPE), Recife, Pernambuco 50670-901, Brazil; 2 Laboratório de Nanotecnologia, Biotecnologia e Cultura de Células (NanoBioCel), Centro Acadêmico de Vitória, Universidade Federal de Pernambuco (CAV/UFPE), Vitória de Santo Antão, Pernambuco 55608-000, Brazil; 3 Faculté de Pharmacie Institut Galien Paris-Saclay - UMR-CNRS 8612, 27048 Université Paris-Saclay, Orsay 91400, France; 4 Laboratory of Cheminformatics (LabChem), Faculdade de Farmácia, Universidade Federal de Goiás (UFG), Goiânia, Goiás 74605-170, Brazil; 5 Laboratório de Biotecnologia Farmacêutica (BioTecFarm), Departamento de Farmácia, Universidade Federal da Paraíba (UFPB), João Pessoa, Paraíba 58051-900, Brazil

**Keywords:** polyisobutylcyanoacyilate, chitosan, fucoidan, levan, C3 pathway activation

## Abstract

Drug-loaded nanosystems are often associated with the advantages of controlled drug release and reduced toxicity. Understanding the activation of the complement system by polysaccharide-coated nanosystems becomes a crucial step in the development of new nanomedicines since the nonactivation of the complement system allows the nanosystems to circulate blood for longer. The aim of this study was to evaluate the activation of the complement system via the C3 protein pathway by polyisobutylcyanoacrylate (PIBCA) nanoparticles coated with polysaccharides (fucoidan, chitosan, or levan) with different surface morphologies. To this end, the nanoparticles were developed using anionic emulsion polymerization (AEP) and redox emulsion polymerization (RREP) techniques were applied to obtain nanoparticles with the same chemical composition but different polysaccharide architectures. The complement system activation studies were carried out using the 2D immunoelectrophoresis technique. Polysaccharide-coated nanoparticles were obtained with sizes ranging from 99.0 ± 0.5 to 659.9 ± 39.0 nm. As expected, the surface charge of the nanoparticles varied as a function of the polysaccharide coating: positive charges for chitosan-NPs, negative charges for fucoidan-NPs, and neutral charges for levan-NPs. According to the results, Chi-NPs did not activate the complement system, while Fuc-NPs and Lev-NPs did, depending on the surface morphology of the polysaccharides. The nanoparticles (Fuc-NPs and Lev-NPs) obtained by the AEP technique were strong complement activators, and those obtained by the RREP technique seemed to induce the formation of aggregates with the C3b protein. The molecular docking results reinforce these findings, highlighting the regions of the polysaccharide interaction with the C3 and C3b proteins. The presence of some chemical groups, such as the sulfate groups present in fucoidan, on the surface of the nanoparticles may contribute to the activation of the complement system.

## Graphical abstract

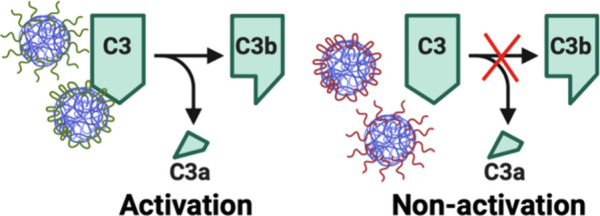

## 1 Introduction

The complement system plays a crucial role in the body’s innate defense against pathogens, being composed of more than 30 proteins hierarchically organized in proteolytic cascades, whose function is to identify the foreign component, activate the release of potent proinflammatory mediators, promote opsonization, and stimulate targeted cell lysis.^1^ There are three pathways of the complement system, namely, the classical, lectin, and alternative pathways, all of which depend on the cleavage of the C3 protein and the production of the C3b protein as a common pathway for the activation of the complement system.^2^ The activation of the complement system by drug-loaded nanoparticles can reduce the nanosystem’s circulation time in the blood, resulting a consequent reduction of the drug’s biological activity.^3^

The structural conformation of polysaccharide molecules on the surface of coated nanoparticles determines their ability to activate the complement system. As previously reported, two different types of polyisobutylcyanoacrylate (PIBCA) nanoparticles coated with dextran or sulfated dextran were obtained using *in situ* polymerization reactions (Figure 1), resulting in the “long buckles” conformation by the anionic emulsion polymerization (AEP) technique and the “brush” conformation by the redox radical emulsion polymerization (RREP) technique.^4–6^ The differences in the conformation of polysaccharides on the surface of nanoparticles are due to methodological differences in the polymerization processes, in which AEP nanoparticles use hydroxyl anions to initiate polymerization, while for RREP nanoparticles, the initiator cerium­(IV) ammonium nitrate is used, which forms redox radicals and is associated with a highly acidic pH that causes the sugar monomers to have a partially open conformation, thereby facilitating the oxidation reaction induced by cerium.^5,6^ These differences in conformation have demonstrated differences in the activation of the complement system.^7,8^

**Figure 1:**
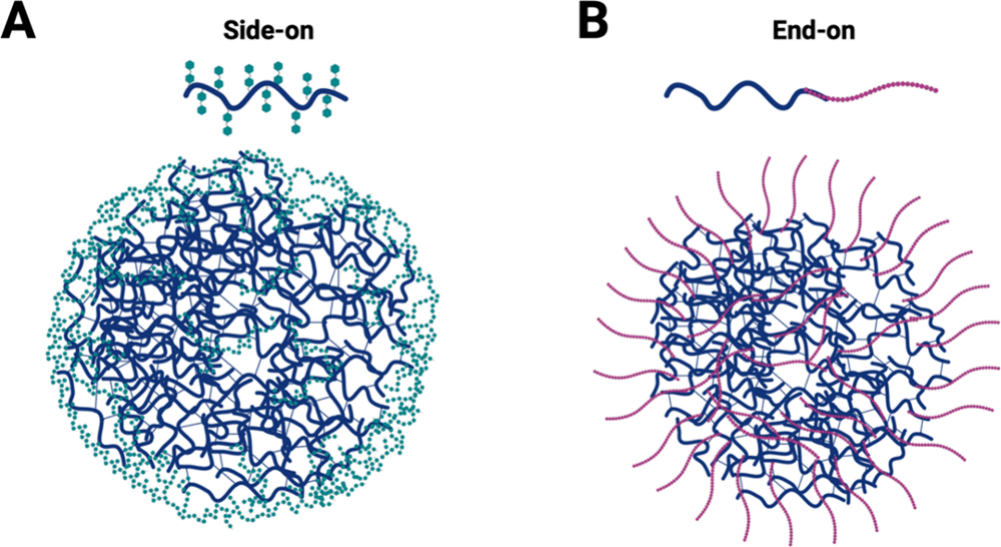
Conformation of long buckles with side-on bonds (A) and brushes with end-on bonds (B) on the surface of the PIBCA nanoparticles. Source: Created by the authors.

The chemical structure of polysaccharide molecules and their arrangement on the surface of nanoparticles influence their interactions with plasma proteins, which can adhere to the nanoparticle surface, forming a protein corona.^3,9^ Corona formation in nanoparticles can induce or inhibit complement activation depending on which proteins form the corona. Interactions with complement proteins imply the activation of complement processes. On the other hand, when there are changes in the chemical conformation of the surface of the nanoparticles, making them more hydrophilic, they appear to drastically decrease complement activation.^10^ The surface charge seems to be a determining factor for the activation of the complement system, as it is believed that nanoparticles with anionic surfaces attract Ca^2+^ ions, which are vital for the activation of the complement system via the classical pathway.^3^

Based on these findings, this study aimed to evaluate the influence of the surface charge and surface conformation of PIBCA nanoparticles coated with polysaccharides on the activation of the complement system. Different polysaccharides (fucoidan, chitosan, and levan) were used for coating the PIBCA NPs, allowing obtaining of two types of PIBCA nanoparticles with “long buckles” and “brush” surface conformations and positive, negative, and neutral surface charges.

## 2 Materials and Methods

### 2.1 Materials

Fucoidan (Fuc) (from *Fucus vesiculosus*, Mw 44 700 Da), levan (Lev) (from *Zymomonas mobilis*, Mw 275.95 kDa), dextran (69 kDa), human serum albumin (HSA, Mw 66 478 Da), nitric acid, and cerium­(IV) ammonium nitrate were obtained from Sigma-Aldrich (USA), and anti-C3 antibody was obtained from Fitzgerald Industries International (San Diego, USA). Water-soluble chitosan (Chi) (Mw 20,000 g/mol) was purchased from Amicogen (Jinju, South Gyeongsang, South Korea). Isobutylcyanoacrylate (IBCA) was obtained from Afinitica (Barcelona, Spain). The human serum was obtained from the Établissement Français du Sang (ESF, agreement 14/EFS/041, Rungis, France) from healthy donors. Figure 2 presents the chemical structures of the different polysaccharides used to obtain the two types of PIBCA nanoparticles with “long buckles” and “brush” surface conformations.

**Figure 2:**
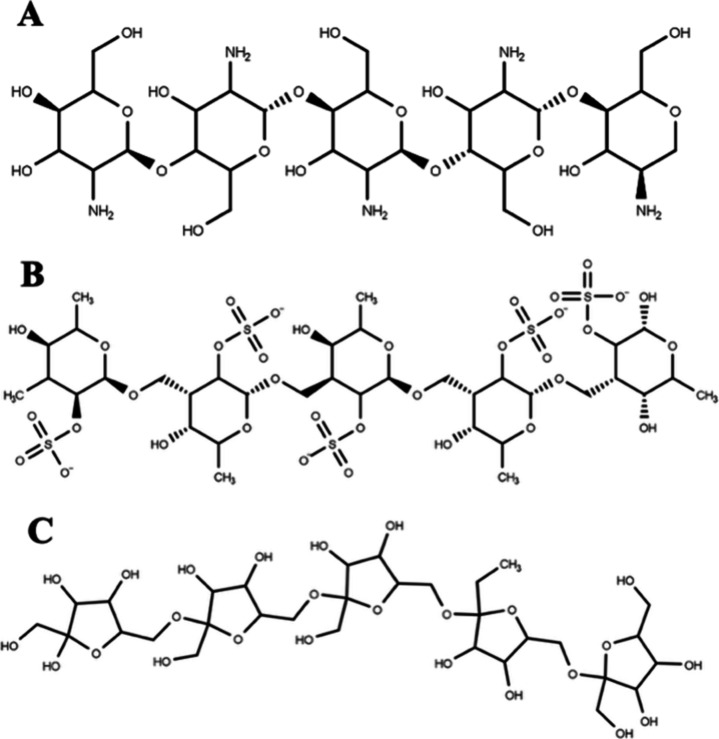
Chemical structures of polysaccharides: chitosan (A), fucoidan (B), and levan (C).

### 2.2 Development of Polysaccharide-Coated PIBCA Nanoparticles

#### 2.2.1 AEP Technique

The nanoparticles (Fuc-NPs-AEP, Chi-NPs-AEP, and Lev-NPs-AEP) were prepared as previously described by Bertholon et al.^4^ and Lira et al.^11^ Briefly, 50 mg of polysaccharide (Fuc, Chi, or Lev) was solubilized in 5 mL of acidified ultrapure water (0.2 M nitric acid). The solution remained under magnetic stirring (1200 rpm), and then, 50 μL of isobutylcyanoacrylate (IBCA) was added, and the agitation was maintained for 1 h at 37 °C.

#### 2.2.2 RREP Technique

The nanoparticles (Fuc-NPs-RREP, Chi-NPs-RREP, and Lev-NPs-RREP) were prepared according to Bertholon et al.^4^ and Lira et al.^11^ For this, 67.8 mg of polysaccharide (Fuc, Chi, or Lev) was weighed and dissolved in 4 mL of 0.2 M nitric acid. The solution was then placed under magnetic stirring at 40 °C with nitrogen bubbling for 10 min. Subsequently, 1 mL of cerium­(IV) ammonium nitrate (0.08 M in 0.2 M nitric acid) and 250 μL of IBCA were added to the solution with vigorous magnetic stirring (1200 rpm). The system was maintained under magnetic stirring for 1 h at 40 °C.

#### 2.2.3 Purification of Nanoparticles

To purify the nanoparticles, the dialysis technique was employed (Spectra-Por membrane 100,000 g/mol molecular weight cutoff (MWCO), Biovalley, Marne-la-Vallée, France) against 1 L of ultrapure water. The water was refreshed twice at 30 min intervals, and subsequently, the system was maintained by stirring at 300 rpm overnight. Nanoparticle dispersions were stored at 4 °C.

### 2.3 Characterization of Nanoparticles

The mean diameter of the nanoparticles, polydispersity index (PDI), and zeta potential (surface charge) were evaluated at 25 °C with a fixed angle of 90° using the light scattering technique on the Zetasizer ZS90 equipment (Malvern Instrument, USA). For the analyses, formulations were diluted in water or sodium chloride (NaCl) 1 mM (1:20) for particle size analysis or zeta potential analysis, respectively.

### 2.4 Fourier-Transform Infrared Spectroscopy Analysis

Fourier-transform infrared spectroscopy (FT-IR) analysis was used to obtain the spectra of nanoparticles in the 4000–400 cm^–1^ range with a resolution of 4 cm^–1^ using an FT-IR/UATR (Spectrum Two, PerkinElmer, Massachusetts, USA).

### 2.5 Interaction of Nanoparticles with HSA and Human Serum

To evaluate whether nanoparticles interact with HSA and human serum, coated nanoparticles (surface concentration of 1000 cm^2^.mL) were incubated with different concentrations of HSA or human serum (0–40 mg/mL) for 1 h under stirring at 37 °C.^12^ After incubation, the zeta potential was evaluated.

### 2.6 Complement System Activation Study

The nanoparticles were evaluated for their capability to activate the complement system via the C3 pathway following the technique proposed by Coty et al.^7^ The nanoparticles (5 mL) were previously concentrated by the counter-dialysis technique^13^ in 0.3% dextran for 22 h, using a 3.5 MWCO membrane (Spectra-por, Biovalley, Marne la Vallée, France). First, nanoparticles at surface concentrations ranging from 25 to 3000 cm^2^.mL were diluted in water (1:2); afterward, saline solution in Veronal buffer supplemented with calcium (VBS^2+^) was added (1:4), and finally, human serum was added (1:4) with a final volume of 50 μL. Samples were then incubated at 37 °C for 1 h. Water and Dex-PIBCA-NPs were used as negative and positive controls, respectively. After incubation, samples were kept on ice until analysis. The specific interactions of nanoparticles with proteins C3 and C3b were evaluated by using two-dimensional (crossed) immunoelectrophoresis. This two-dimensional immunofluorescence technique allows the quantification of protein mixtures, in which the proteins are separated by gel electrophoresis in the first dimension and, in the second dimension, the separated proteins come into contact, by right-angle electrophoresis, with a gel layer containing anti-C3 antibodies.

### 2.7 *In Silico* Approaches

#### 2.7.1 Structure Collection and Prioritization

All the 3D structures have been compiled from human C3 and C3b proteins from the Protein Data Bank (PDB), ensuring a resolution not exceeding 4.0 Å, within the scope of our study (see Table S1 for details). To identify key conformations for further investigation, we performed principal component analysis (PCA) and hierarchical clustering analysis (HCA) utilizing the Bio3D package.^14^ These analyses were conducted on the entire set of 3D structures of C3 and C3b complexes to elucidate interconformer relationships. Subsequently, the most representative conformations were prioritized for further analysis.

#### 2.7.2 Binding Site Identification

The identification of the most representative C3 and C3b complex conformations involved an additional step using the FTMap server^15^ to pinpoint potential binding sites. This server employs a comprehensive approach, employing 16 small organic molecules with diverse shapes and polarities, systematically distributed in a dense grid surrounding the C3 and C3b structures.^15^

#### 2.7.3 Protein Preparation

The prioritized protein conformations were imported into Maestro workspace v.9.3 (Schrödinger, LCC, New York, 2012) and processed using the Protein Preparation Wizard. In this step, hydrogen atoms were added to the proteins, while bond orders and formal charges were adjusted. Furthermore, the Epik program^16^ was employed to predict the protonation states (p*K*_a_) of polar amino acids at pH = 7.4 ± 0.5, whereas the PROPKA v.3.1 ^17^ was used to optimize the hydrogen orientations.

#### 2.7.4 Ligand Preparation

The 2D structures of Fuc, Lev, and Chi were imported into the Maestro workspace v.9.3 (Schrödinger, LCC, New York, 2012) and prepared under neutral pH conditions (7.4 ± 0.5) using LigPrep v.2.5. Then, each sugar underwent geometric optimization through the OPLS_2005 force field,^18^ aiming to achieve the most energetically favorable conformations.

#### 2.7.5 Ensemble Docking Protocol

Five grid boxes were established in *x*, *y*, and *z* coordinates of C3 and C3b complexes using the receptor grid generation panel of Glide v.5.8.^19^ Details of the grid boxes are shown in Table S2. They were generated to assess the most suitable conformations of the binding sites for the docking. Then, molecular docking calculations were performed in the Maestro workspace Glide v.5.8 ^19,20^ using standard precision (SP) mode. The poses were scored using GlideScore, Glide Energy, and Glide Emodel scoring functions^19,20^ and further optimized using the OPLS_2005 force field,^21^ which ensures that the poses are refined locally.

#### 2.7.6 MM/GBSA Rescoring

To rescore ensemble docking complexes, we implemented molecular mechanics/generalized Born surface area (MM/GBSA) analysis using Prime v.3.1 (Schrödinger, LCC, New York, 2012). MM/GBSA is an approach that combines a detailed representation of molecular interactions (MM) with a simplified treatment of solvation effects (GB), offering a reliable estimation of the binding free energy. This technique is particularly valuable for evaluating binding affinity in ligand-protein systems and for evaluating the distinct contributions of individual components to the overall binding energy. The general equation for the MM/GBSA calculation is as follows:

$$\Delta G_{\mathrm{bind}}=\Delta G_{\mathrm{MM}}+\Delta G_{\mathrm{GB}}-\mathrm{TS}$$

Here, Δ*G*_bind_ represents the binding free energy; Δ*G*_MM_ is the contribution from molecular mechanics, involving terms like bond energy, torsional energy, van der Waals, and electrostatic interactions; Δ*G*_GB_ accounts for the contribution from Generalized Born solvation, taking into consideration the solvation effects based on surface area and polarity; and TS is the entropic correction, considering changes in entropy during the binding process.^22^

## 3 Results and Discussion

### 3.1 Polysaccharide-Coated PIBCA Nanoparticles

The nanoparticles were obtained by AEP and RREP techniques using the Fuc, Chi, and Lev for coating and characterized regarding particle size, PDI, and zeta potential (Table 1). The particle size of nanoparticles (DLS measurement graphs in Figure S1) varied from 99.0 ± 0.5 to 659.9 ± 39.0 nm, which is related to the obtaining technique as well as the polysaccharide coating. Homogeneous-sized nanoparticles were obtained (PDI < 0.3).

**Table 1:** Characterization of Polysaccharide-Coated PIBCA Nanoparticle Suspensions

method	formulation	size (nm)	PDI	zeta potential (mV)
AEP^a^	Fuc-NPs	349.2 ± 5.8	0.05 ± 0.04	–43.1 ± 1.2
	Chi-NPs	99.0 ± 0.5	0.16 ± 0.01	+52.7 ± 1.3
	Lev-NPs	422.0 ± 16.1	0.33 ± 0.03	–0.7 ± 0.1
RREP^a^	Fuc-NPs	150.9 ± 2.5	0.20 ± 0.03	–25.8 ± 1.6
	Chi-NPs	296.6 ± 1.1	0.05 ± 0.02	+57.1 ± 2.7
	Lev-NPs	659.9 ± 39.0	0.07 ± 0.05	–5.2 ± 0.4

a AEP = anionic emulsion polymerization; RREP = redox radical emulsion polymerization.

As expected, the surface charge of nanoparticles varied as a function of the chemical structure of the polysaccharide coating: positive charges for Chi-NPs due to Chi’s amine groups,^23–26^ negative charges for Fuc-NPs due to Fuc sulfate groups,^11,26–28^ and neutral charges for Lev-NPs.^26,29,30^ In the case of Fuc coating, the difference between the surface charge of the nanoparticles obtained by the RREP (around −25.8 ± 1.6 mV) and AEP (around −43.1 ± 1.2 mV) may be related to the disposition of sulfate groups on the surface of the nanoparticles, as previously reported.^11,28^ The different chemical structures of the polysaccharides impacted both the size of the nanoparticles and the surface charge. Lev, which has a longer chemical chain (Mw 275 950 Da), was larger in size compared to the others (Fuc Mw 44,700 Da and Chi Mw 20,000 Da), corroborating the findings presented by Cavalcanti et al.^26^

### 3.2 FT-IR Analysis of Polysaccharide-Coated PIBCA Nanoparticles

FT-IR data are listed in Table 2. The spectra of the Fuc-NPs-AEP and Fuc-NPs-RREP (Figure 3) were also similar to those obtained by Cavalcanti et al.,^27,31^ with an absorption band at 3469 cm^–1^ (Fuc-NPs-AEP) and 3418 cm^–1^ (Fuc-NPs-RREP) referring to the O–H bond, at 1746 cm^–1^ (Fuc-NPs-AEP) and 1748 cm^–1^ (Fuc-NPs-RREP) referring to the C=O bond, and at 1247 and 1166 cm^–1^ referring to CO. The bonds of the sulfate groups characteristic of Fuc are in the region of 1470 and 1384 cm^–1^ referring to the S=O bond and 970 cm^–1^ referring to the SO bond.^32,33^

**Table 2:** Chemical Groups and Wavenumber of the Absorption Bands in the FT-IR Spectra of the Samples

method	samples	O–H (cm^–1^)	C–H (cm^–1^)	C=O (cm^–1^)	amide I (cm^–1^)	amide II (cm^–1^)	CH_3_ (cm^–1^)	S=O (cm^–1^)		amide III (cm^–1^)	C–N (cm^–1^)	C–O (cm^–1^)		C–O–C (cm^–1^)	S–O (cm^–1^)	furanose (cm^–1^)
AEP	Fuc-NPs	3469	2979	1746				1470	1384			1247	1166		970	
	Chi-NPs	3358	2967	1746	1626	1526	1470			1372	1247			1018		
	Lev-NPs	3359	2966	1750								1262		1018		932
RREP	Fuc-NPs	3418	2964	1748				1470	1384			1246	1169		969	
	Chi-NPs	3366	2972	1750	1631	1523	1471			1380	1247			1018		
	Lev-NPs	3371	2964	1742								1243		1075		942

**Figure 3:**
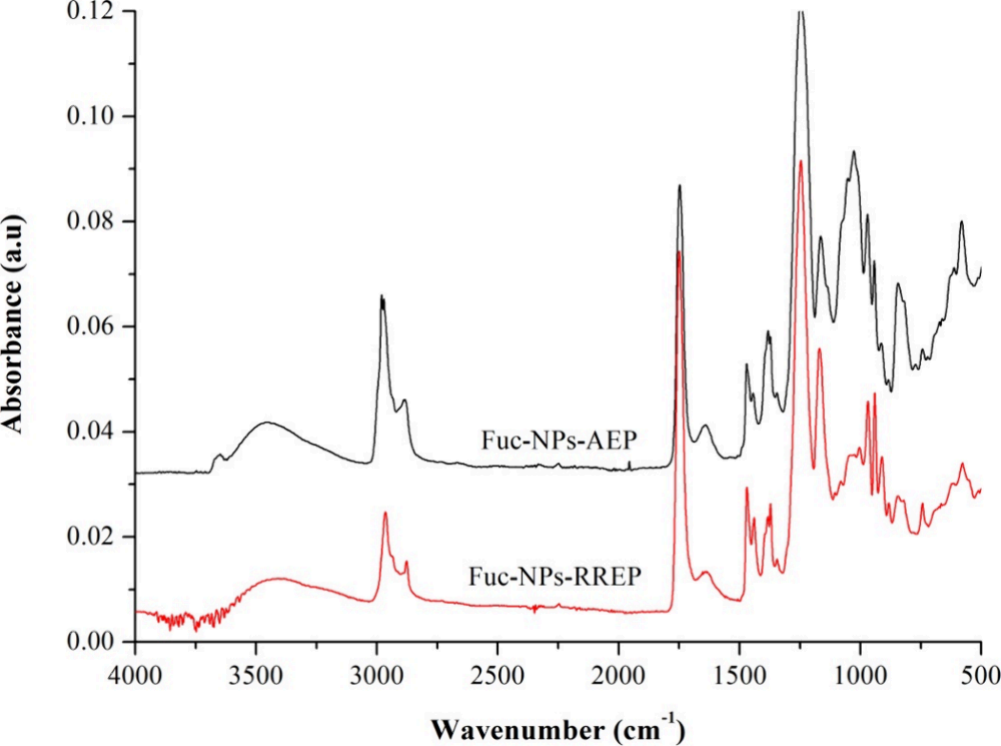
FT-IR spectra of Fuc-NPs-AEP (black line) and Fuc-NPs-RREP (red line).

The absorption spectra of the Chi-NPs (Figure 4) are in concordance with the study by Pramanik et al.,^34^ Queiroz et al.,^35^ Vijayalakshmi et al.,^36^ Tran et al.,^37^ and Guarnizo-Herrero et al.,^38^ which evaluated the absorption spectrum of Chi. An absorption band can be seen at 3358 cm^–1^, which is related to the presence of N–H and O–H stretching. The absorption bands around 2967 and 2879 cm^–1^ may be related to symmetric and asymmetric C–H stretching, respectively. The band around 1746 cm^–1^ refers to the C=O stretching of amide I and at 1372 cm^–1^ refers to the C=O stretching of amide III. Another absorption band at 1526 cm^–1^ may be related to the N–H bending of amide II, and the absorption band at 1626 cm^–1^ may be related to the N–H bending of the primary amide. The absorption band at 1080 cm^–1^ is related to the presence of the C–O–C group present in water-soluble chitosan, corroborating the study by Yusharani et al.^39^

**Figure 4:**
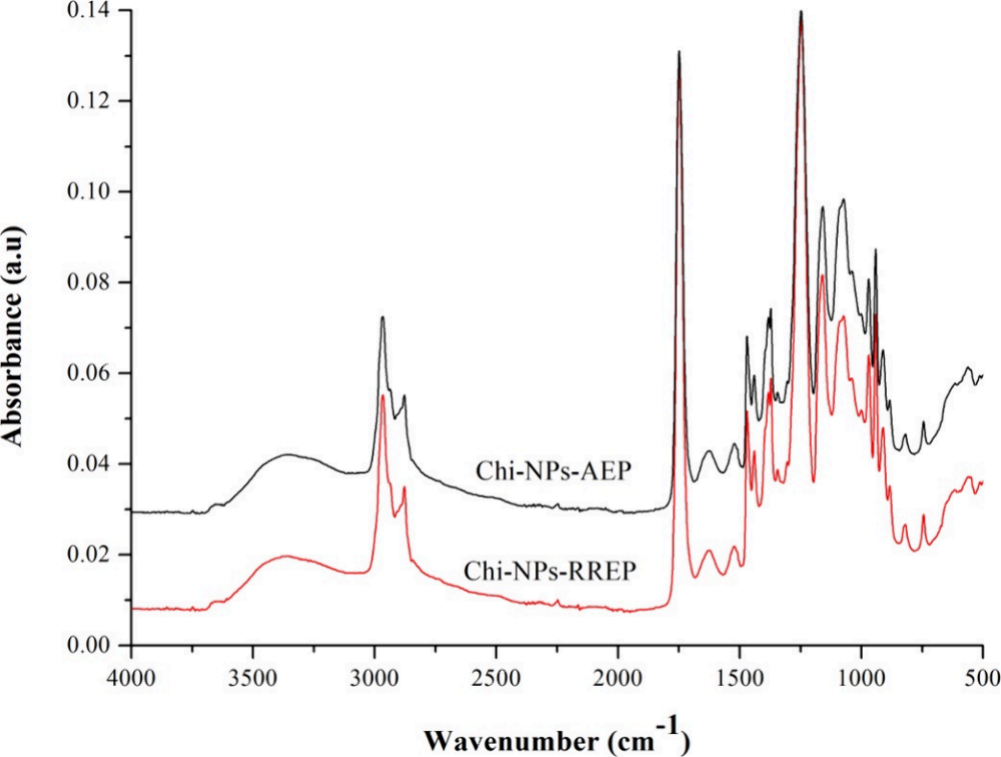
FT-IR spectra of Chi-NPs-AEP (black line) and Chi-NPs-RREP (red line).

In the absorption spectrum of the nanoparticles coated with Lev (Figure 5), absorption bands, such as those in the spectrum of Fuc and Chi nanoparticles, are observed around 3359 cm^–1^ (Lev-NPs-AEP) and 3371 cm^–1^ (Lev-NPs-RREP) relating to O–H stretching, as well as absorption bands around 2966 and 2877 cm^–1^ relating to symmetrical and asymmetrical C–H stretching, respectively. Specifically, from levan, we can observe an absorption band at 1018 cm^–1^ (Lev-NPs-AEP) and 1075 cm^–1^ (Lev-NPs-RREP), which is related to the stretching vibration of the glycosidic bond (C–O–C) in pyranose or furanose, which is characteristic of carbohydrates, and at 932 cm^–1^ (Lev-NPs-AEP) and 942 cm^–1^ (Lev-NPs-RREP), it is related to furanose. These bands present in the nanoparticle spectra indicate the presence of Lev, corroborating the study by Cavalcanti et al.^26^ and Silva et al.^29^ Kim and Chung^40^ report that modification of Lev with cerium oxide increased the maximum intensity of the hydroxyl functional group, which was also observed in this study in Lev-NPs-RREP, in which cerium­(IV) ammonium nitrate is used.

**Figure 5:**
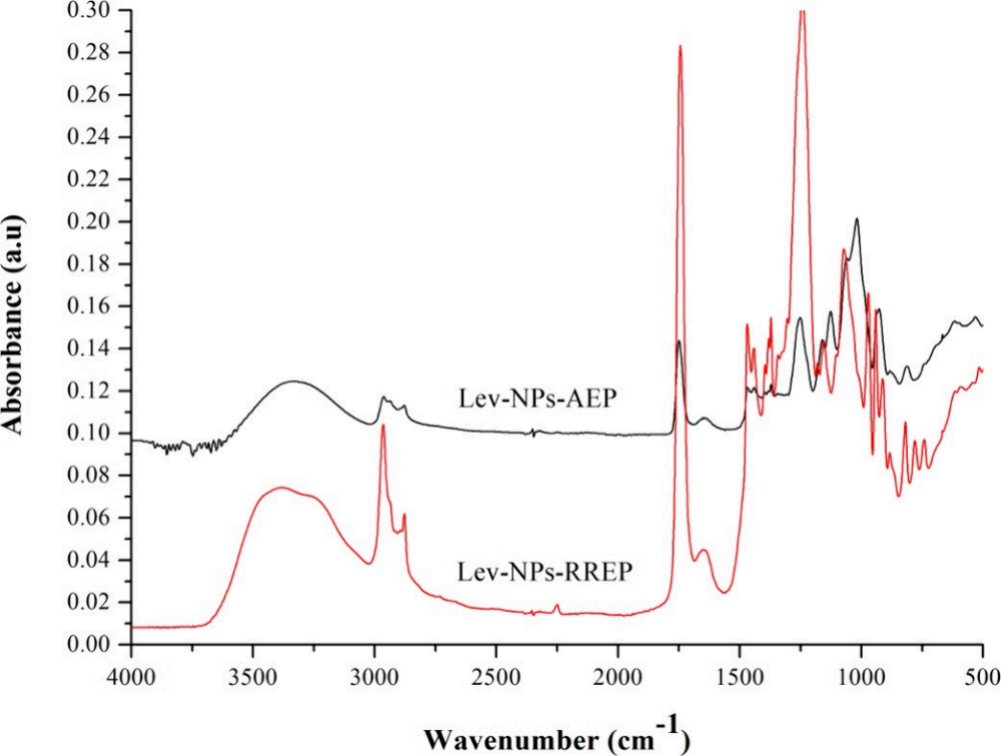
FT-IR spectra of Lev-NPs-AEP (black line) and Lev-NPs-RREP (red line).

### 3.3 Protein Adsorption on the Surface of Polysaccharide-Coated PIBCA Nanoparticles

The study of protein adsorption on the surface of nanoparticles showed that the HSA interacted with Fuc-NPs after exposure to different concentrations of HSA (Figure 6A,B, blue line), with significant changes in the charge of the nanoparticles from −25.8 ± 1.6 to −35.1 ± 0.5 mV for Fuc-NPs-RREP and small changes from −43.1 ± 1.2 to −42.3 ± 0.1 mV for Fuc-NPs-AEP. On the other hand, when we compared the charge of the Fuc-NPs after exposure to human serum (Figure 6A,B, green line), a slight reduction in negative charge was observed, from −25.8 ± 1.6 to −21.4 ± 1.0 mV (Fuc-NPs-RREP) and a significant reduction for Fuc-NPs-AEP (−43.1 ± 1.2 to −25.0 ± 0.7 mV), which suggests the binding of plasma proteins on the surface of the nanoparticles. These results were like those presented by Cavalcanti et al.^26,27^

**Figure 6:**
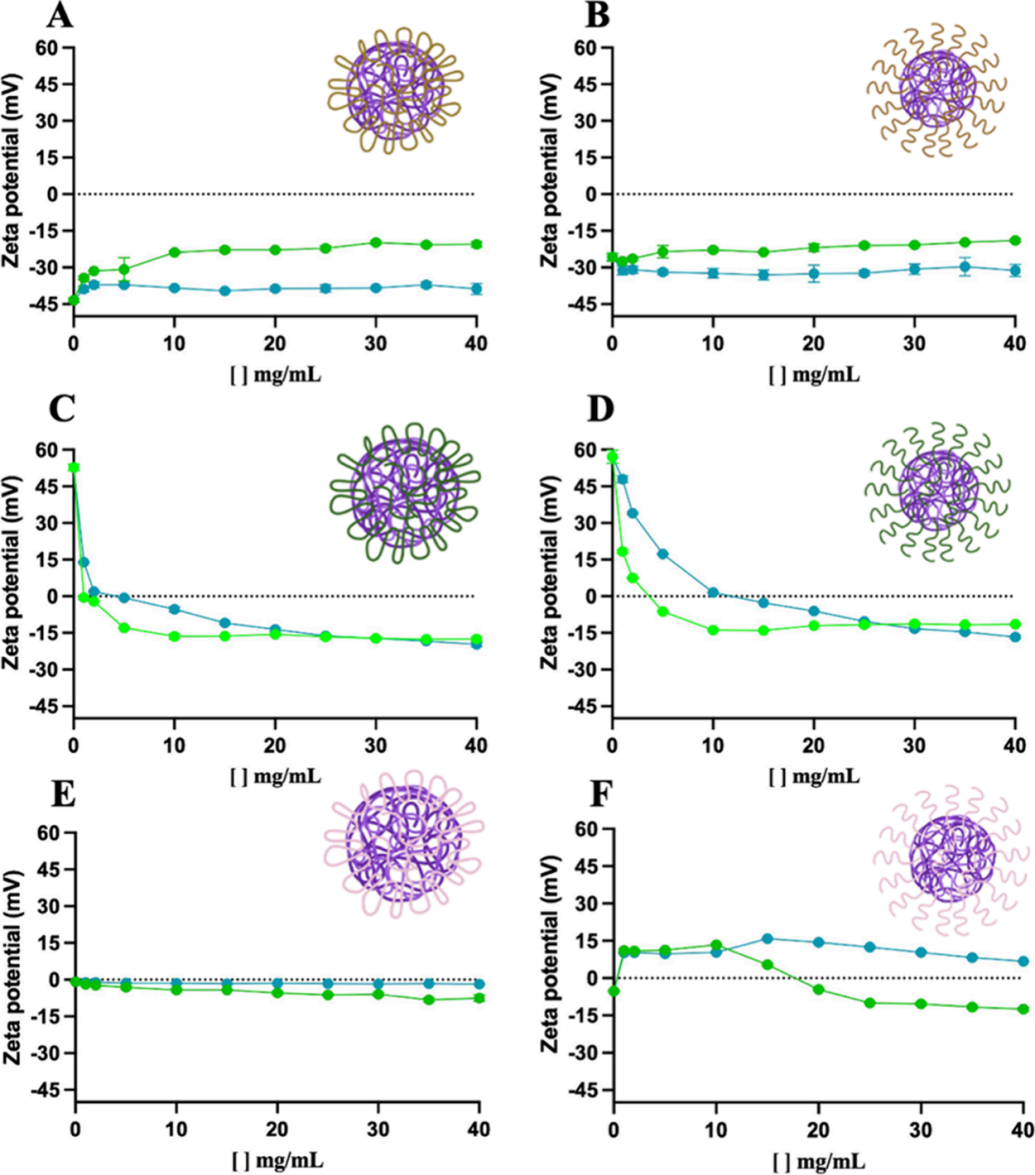
Interaction between HSA (blue line) and human serum proteins (green line) with the nanoparticles Fuc-NPs-AEP (A), Fuc-NPs-RREP (B), Chi-NPs-AEP (C), Chi-NPs-RREP (D), Lev-NPs-AEP (E), and Lev-NPs-RREP (F). The *X*-axis represents [HSA] or [serum proteins].

Regarding Chi-NPs (Figure 6C,D, blue line), the change in surface charge is very evident when the nanoparticles are exposed to HSA, going from a charge of +52.7 ± 1.3 to −16.6 ± 0.6 mV for Chi-NPs-RREP and from +57.1 ± 2.7 to −19.7 ± 0.8 mV for Chi-NPs-AEP. These results suggest a strong interaction between Chi-NPs and HSA and indicate possible formation of the protein corona.^12^ The results with human serum were also like the interaction profile of these nanoparticles with HSA, showing a probable strong attraction of HSA to the Chi-NPs (Figure 6C,D, green line).

In relation to the Lev-NPs (Figure 6E,F), no significant change was observed in the charge of the nanoparticles when exposed to HSA. However, when the samples exposed to human serum were evaluated, two profiles were observed: (i) Lev-NPs-AEP nanoparticles (Figure 6E) showed no significant difference in the charge of the nanoparticles; but (ii) Lev-NPs-RREP (Figure 6F) appeared to interact with molecules present in the serum. These differences between the nanoparticles may be related to their surface morphology, which can impact on the differences in interactions with biological media, as was observed in the study by Lira-Nogueira et al.^28^

These changes in the charge of Fuc-NPs and Lev-NPs when exposed to human serum may indicate the affinity of interaction of these nanoparticles with other proteins present in serum, and this affinity may be different for the types of nanoparticles (Fuc-NPs-AEP or Fuc-NPs-RREP), since these have different surface conformation. According to Coty et al.,^41^ differences in the surface conformation of nanoparticles allow them to have different affinities for plasma proteins, and although both nanoparticles had similar results in terms of surface charge change, perhaps, different proteins are binding to the nanoparticles. Also, it is possible to observe that the dextran nanoparticles obtained by the RREP technique presented higher adsorption of the proteins Insulin B chain, aprotinin, apo-transferrin, and immunoglobulin G, while the dextran nanoparticles obtained by the AEP technique showed higher adsorption of the proteins apo-transferrin, immunoglobulin G, albumin, insulin B chain, and aprotinin. In addition, according to Labarre et al.,^42^ the sulfated dextran nanoparticles showed higher adsorption of the opsonin proteins C3 and C3b compared with the dextran nanoparticles. This high affinity of these opsonin proteins may also be occurring with Fuc nanoparticles since it is also a sulfated polysaccharide.

### 3.4 Complement System Activation Study

For the complement system activation study (Figure 7), the nanoparticles were concentrated using the counter-dialysis technique proposed by Vauthier et al.^13^ This technique proved effective in increasing the surface concentration of Fuc-NPs-AEP, Chi-NPs-RREP, Chi-NPs-AEP, Lev-NPs-AEP, and Lev-NPs-RREP, promoting a 10-fold increase in concentration (see Table S3) after 22 h, with a reduction in the volume of water in the dispersion. It should be noted that the physicochemical characteristics of the nanoparticles were maintained with no significant variation in size, PDI, or zeta potential (see Table S3).

**Figure 7:**
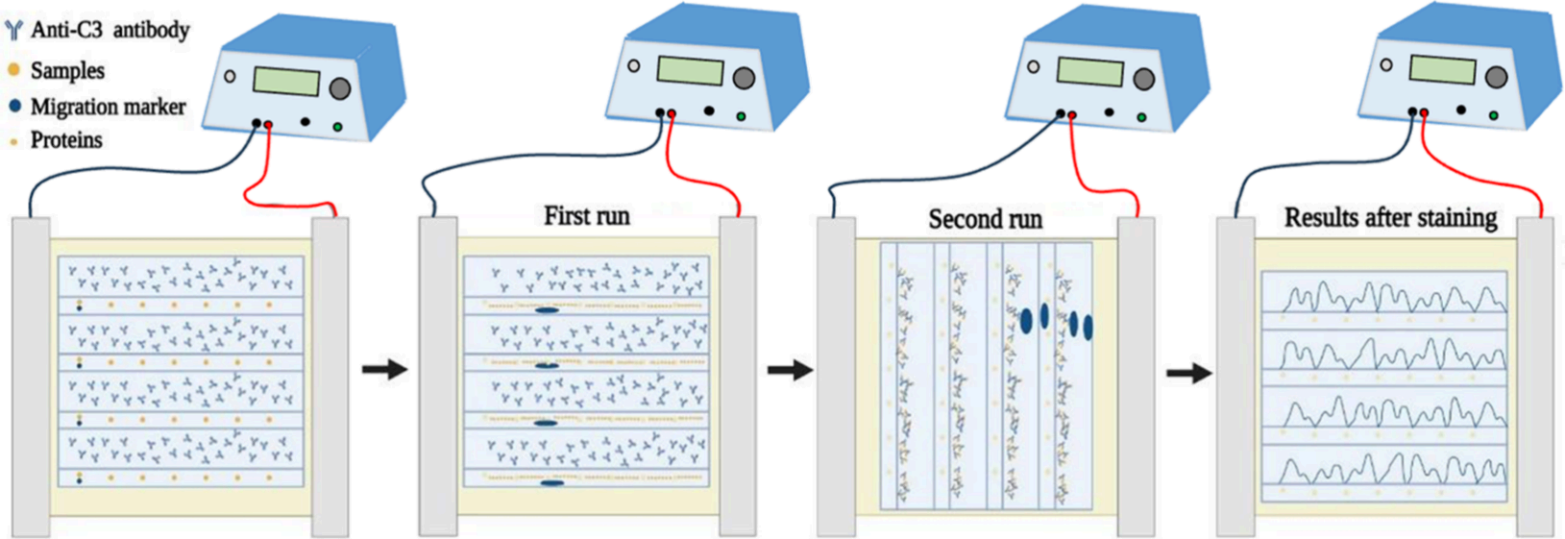
2D electrophoresis runs for analysis of complement system activation studies. In the first run, the proteins are separated according to size; in the second run, the samples will interact with the anti-C3 antibody, where the anti-C3 present in the gel will select the C3 proteins; the interaction of the proteins with the anti-C3 will produce peaks, in which the first peak refers to the C3 protein and the second to the C3b protein, which can be visualized after staining. Source: Created by the authors.

Fuc-NPs-AEP (Figure 8, green line) activated the complement system in all tested concentrations (25–3000 cm^2^.mL). These results corroborate the results obtained by Coty et al.^7,8^ who observed that dextran nanoparticles (neutral surface charges) with different densities obtained by the AEP technique activate the complement system. The results with Fuc-NPs-RREP (Figure 8, blue line) also showed activation of the complement system, but at higher concentrations (2000 to 3000 cm^2^.mL), these nanoparticles induced a reduction in the concentration of C3b (see Figure 8, M–O, blue box). These results may indicate that the C3b protein may be adsorbing to the surface of the nanoparticles and forming aggregates, thereby reducing the concentration of this protein. These results agree with the study by Labarre et al.^42^ who observed that sulfated dextran nanoparticles reduced the concentration of the C3 and C3b proteins due to the high adsorption of these proteins on the surface of the nanoparticles. This interaction of the C3 and C3b proteins with the nanoparticles may be related to the presence of a sulfate group.

**Figure 8:**
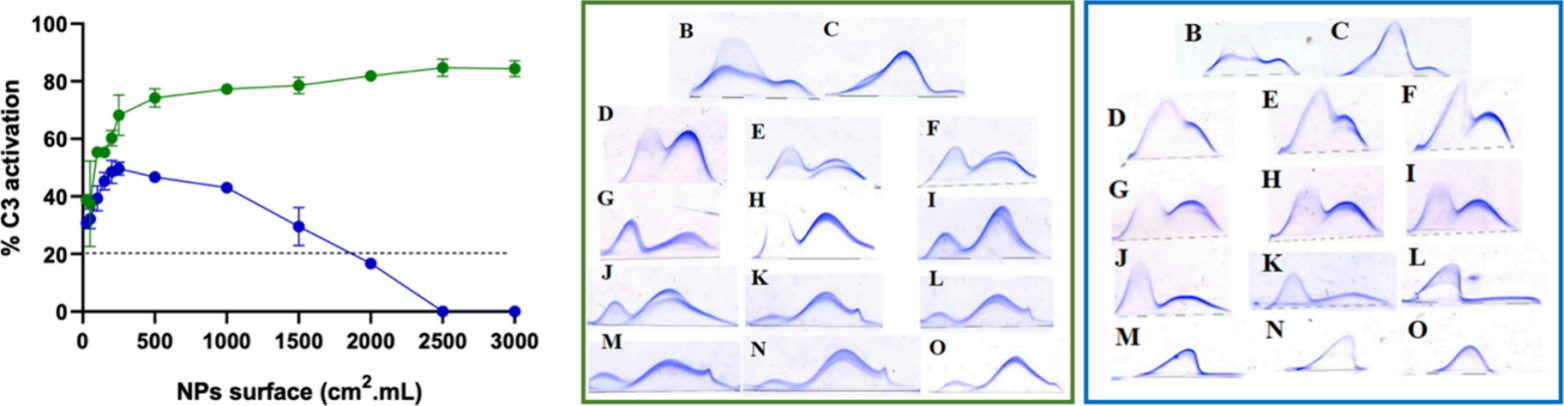
Complement activation profile of Fuc-NPs-AEP (green line) and Fuc-NPs-RREP (blue line). Images of the peaks referring to the negative (B) and positive (C) controls. Peaks of Fuc-NPs-AEP (green box) and Fuc-NPs-RREP (blue box) at surface concentrations of 25 cm^2^.mL (D), 50 cm^2^.mL (E), 100 cm^2^.mL (F), 150 cm^2^.mL (G), 200 cm^2^.mL (H), 250 cm^2^.mL (I), 500 cm^2^.mL (J), 1000 cm^2^.mL (K), 1500 cm^2^.mL (L), 2000 cm^2^.mL (M), 2500 cm^2^.mL (N), and 3000 cm^2^.mL (O).

About the Chi-NPs-AEP (Figure 9, green line), it was observed that, unlike the Fuc-NPs-AEP, replacing Fuc with Chi reduces the activation of the complement system. Chi does not seem to activate the complement system at lower concentrations but activates it discretely at higher concentrations (1000 to 3000 cm^2^.mL), which favors the use of these nanoparticles in intravenous administration, allowing them to remain in the body for longer. Wang et al.^43^ evaluated the concentration of C3a and C5a after incubating carboxymethyl chitosan (CMC) nanoparticles in plasma. The authors observed that these nanoparticles did not influence the concentrations of C3a and C5a, indicating that they are unable to activate the complement system. Although the chemical structure of CMC differs from that of water-soluble chitosan, the results were similar to those presented in this study. These results corroborate those obtained in this study. Of note, Chi-NPs-RREP (Figure 9, blue line) appear to activate more than Chi-NPs-AEP at higher concentrations (2000–3000 cm^2^.mL). Moreover, it was observed that replacing Fuc with Chi reduces the level of activation of the complement system. This difference may be related to structural factors in the surface morphologies of these nanoparticles.

**Figure 9:**
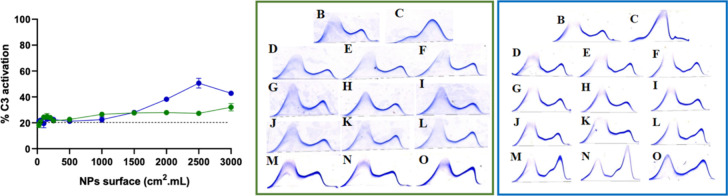
Complement activation profile of Chi-NPs-AEP (green line) and Chi-NPs-RREP (blue line). Images of the peaks referring to the negative (B) and positive (C) controls. Peaks of Chi-NPs-AEP (green box) and Chi-NPs-RREP (blue box) at surface concentrations of 25 cm^2^.mL (D), 50 cm^2^.mL (E), 100 cm^2^.mL (F), 150 cm^2^.mL (G), 200 cm^2^.mL (H), 250 cm^2^.mL (I), 500 cm^2^.mL (J), 1000 cm^2^.mL (K), 1500 cm^2^.mL (L), 2000 cm^2^.mL (M), 2500 cm^2^.mL (N), and 3000 cm^2^.mL (O).

According to the results obtained with the Lev-NPs-AEP (Figure 10, green line), these nanoparticles are strong activators of the complement system, activating at all of the concentrations tested. Figure 10 (blue line) presents the results of complement system activation of Lev-NPs-RREP. It was observed that these nanoparticles activated the complement system, and as the concentration increased from 1000 cm^2^.mL, there was a 0% reduction in the concentration of the C3b protein. As previously indicated for Fuc-NPs-RREP, this may be related to the aggregation of C3b on the surface of the nanoparticles, with only one peak referring to C3 in Figure 10K (blue box). Another factor that may contribute to this aggregation of C3b on the surfaces of Lev-NPs-RREP may be due to the increase in hydroxyl groups, due to the synthesis reaction of the nanoparticles with cerium­(IV) ammonium nitrate, as observed in the FT-IR results and as suggested by Kim and Chung.^40^ According to the study by Stark et al.,^44^ Lev was a strong activator of the complement system in rabbits.

**Figure 10:**
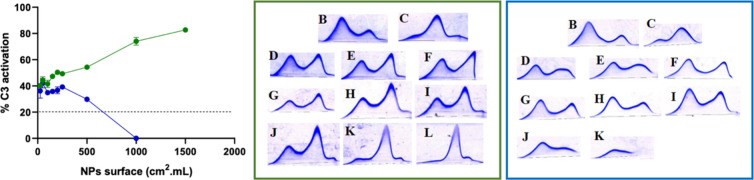
Complement activation profile of Lev-NPs-AEP (green line) and Lev-NPs-RREP (blue line). Images of the peaks referring to the negative (B) and positive (C) controls. Peaks of Lev-NPs-AEP (green box) and Lev-NPs-RREP (blue box) at surface concentrations of 25 cm^2^.mL (D), 50 cm^2^.mL (E), 100 cm^2^.mL (F), 150 cm^2^.mL (G), 200 cm^2^.mL (H), 250 cm^2^.mL (I), 500 cm^2^.mL (J), 1000 cm^2^.mL (K), and 1500 cm^2^.mL (L).

### 3.5 Structural Insights for Complement System Activation

Ensemble molecular docking was employed as a qualitative tool to identify potential recognition regions between polysaccharides and the C3/C3b domains. Initially, a set of 14 C3 or C3b X-ray structures was retrieved from the PDB database. Subsequently, a structural interconformer analysis of these proteins was performed using PCA and HCA to prioritize the most representative protein conformations for prospective investigations. These outcomes guided the selection of five PDB structures: 7ZGK, 3G6J, 5FO8, 6RU5, and 7ZGJ (see Figure 11A,B). All of the PDB IDs used to perform the PCA and HCA can be found in Table S2.

**Figure 11:**
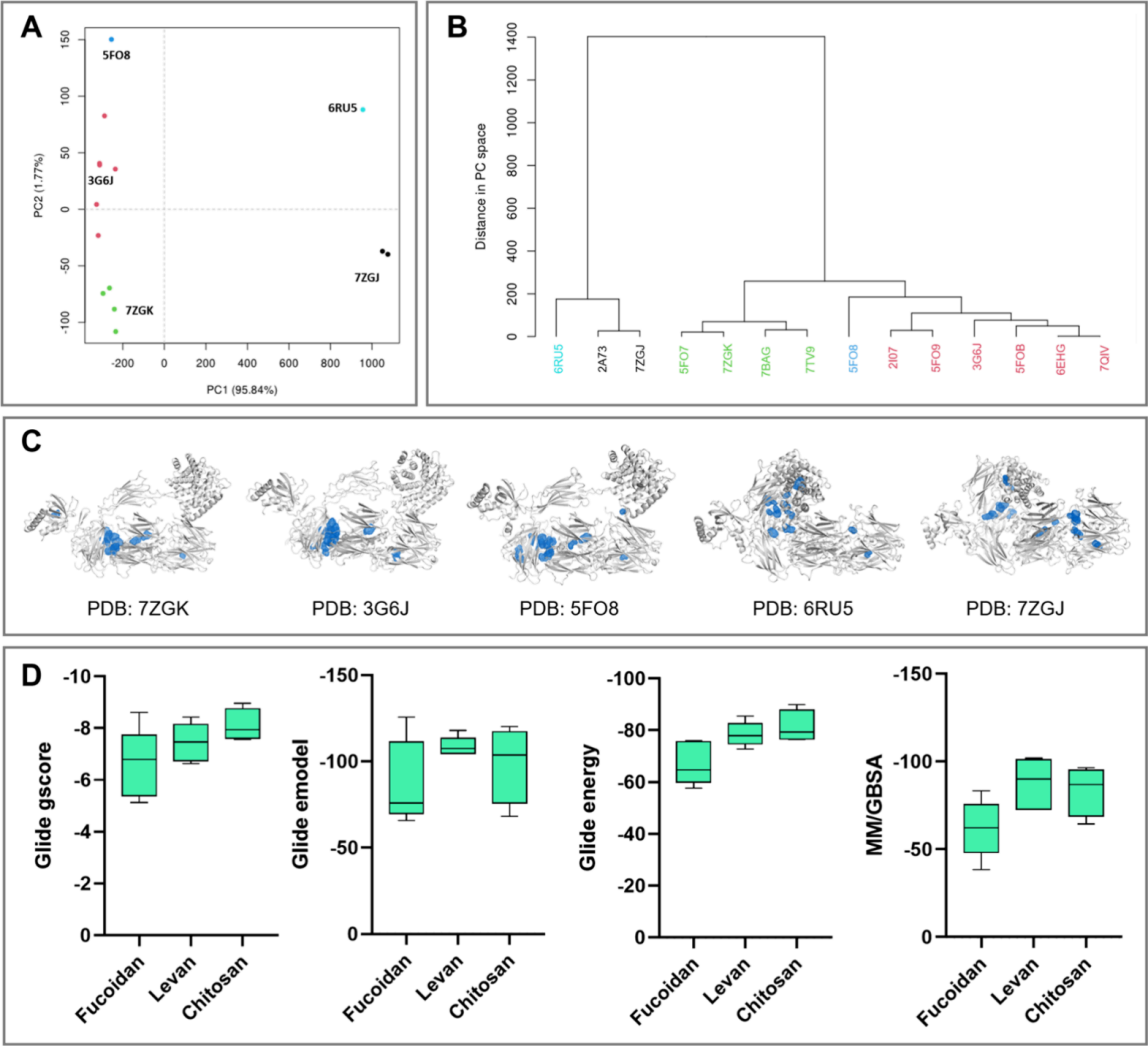
C3 and C3b structural analysis and molecular docking statistical analysis. (A) PCA (principal component analysis). (B) HCA (hierarchical component analysis). (C) Hot spot analysis of the selected conformations, the blue mesh indicates the possible hot spots. (D) Docking score statistical results.

Subsequently, a chemical probe mapping approach was conducted using the FTmap server to identify potential hot spots within the five selected conformations (Figure 11C). This comprehensive analysis prominently pinpointed binding pockets within the TED (Thioester-containing domain), MG8 (Macroglobulin domain), and CUB^45^ for C3 proteins (PDB IDs: 6RU5 and 7ZGJ) and MG2, MG3, MG6, MG7, and MG8 domains^46^ of C3b proteins (PDB IDs: 7ZGK, 3G6J, and 5FO8). Importantly, the conformations demonstrated comparable distributions of hot spots, culminating in the consensus identification of binding sites within the C3 and C3b structures. Subsequently, to elucidate the structural basis of interactions involving Fuc, Lev, and Chi, an ensemble docking investigation was strategically focused on the identified hot spots within the five selected C3 and C3b conformations.

Following our previous experimental analyses (depicted in Figure 11D), the ensemble docking study showed that Lev (Glide gscore = −7.4 ± 0.5 kcal/mol, Glide emodel = −108.7 ± 3.7 kcal/mol, Glide energy = −78.5 ± 3.1 kcal/mol) and Chi (Glide gscore = −8.1 ± 0.4 kcal/mol, Glide emodel = −98.9 ± 15.4 kcal/mol, Glide energy = −81.2 ± 4.3 kcal/mol) exhibited superior docking scores compared to Fuc (Glide gscore = −6.6 ± 1.0 kcal/mol, Glide emodel = −87.6 ± 18.8 kcal/mol, Glide energy = −67.2 ± 6.7 kcal/mol). These findings were additionally validated through rescored analyses based on MM/GBSA, revealing average Δ*G* values of −61.9 ± 10.8, −87.5 ± 11.7, and −83.6 ± 9.8 kcal/mol for Fuc, Lev, and Chi, respectively. Although Lev and Chi displayed more favorable docking scores than Fuc, these results reflect local binding propensities under simplified conditions and should not be interpreted as predictors of complement activation, which is governed by collective, multivalent, and surface-mediated effects.

The docking study elucidates the intricate interactions between nanoparticles and proteins. Figure 12A illustrates the salt-bridge interactions formed by sulfate groups present in Fuc with Lys940, Lys1478, and Lys1436 of C3. However, the stability of this interaction is enhanced by hydrogen bonds with Ala1437, Ile1004, Asn939, and Lys1478. Additionally, Figure 12B highlights the interaction with C3b, demonstrating a robust connection through salt-bridge interactions with Lys1478 and Lys608. Simultaneously, the interaction is reinforced by hydrogen bonds with Val228, Gln340, Lys225, Glu223, Gln203, Lys608, and Glu342. In summary, Fuc exhibits interactions with both C3 and C3b with a preference for binding to C3. The 3D diagram visually confirms this affinity, consistent with the docking scores presented in Figure 12D.

**Figure 12:**
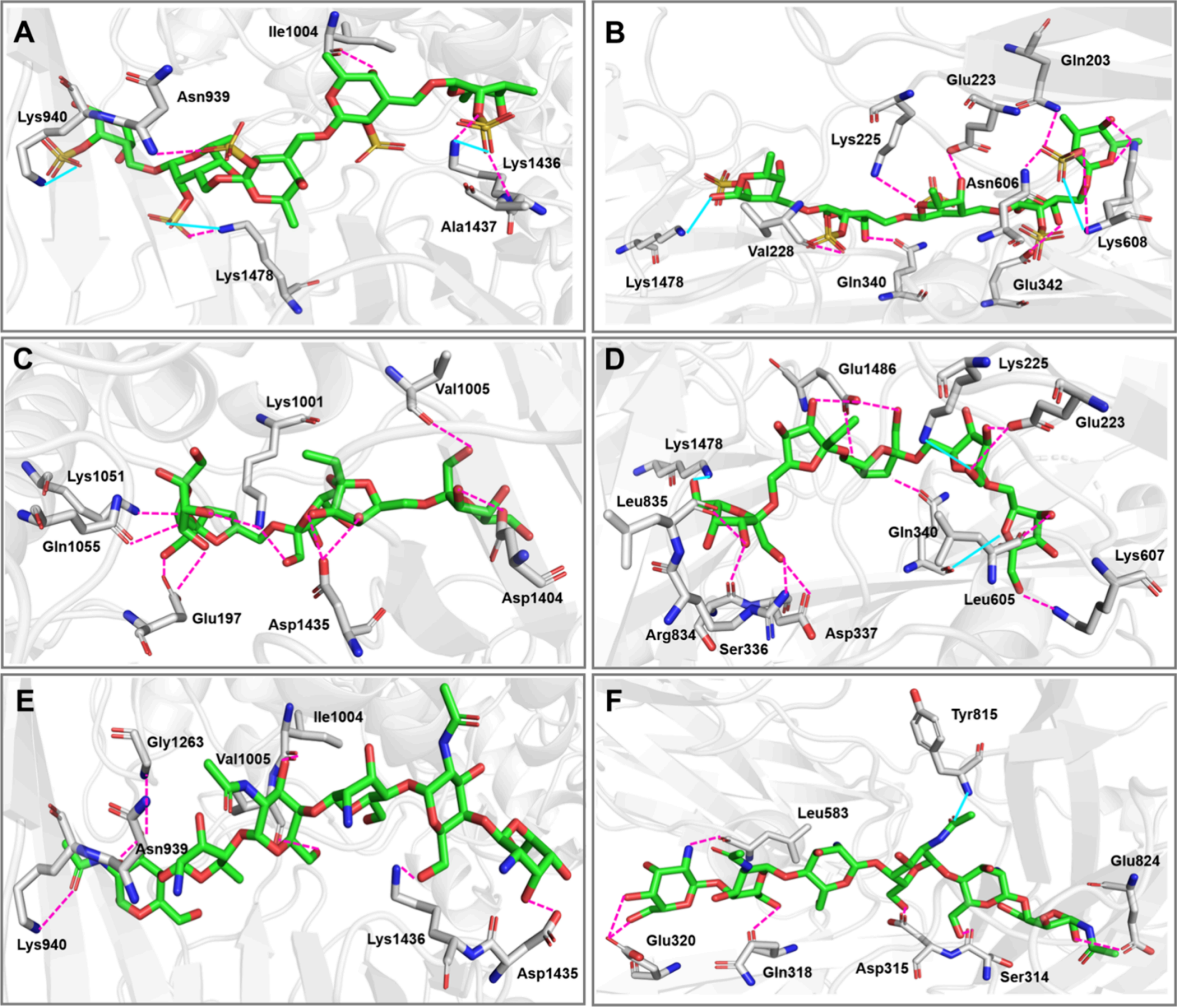
3D molecular docking interaction diagrams. (A, C, and E) 3D diagrams of Fuc, Lev, and Chi interacting with C3 structures (PDB IDs: 7ZGJ, 6RU5, and 7ZGJ, respectively); (B, D, and F) 3D diagram of Fuc, Lev, and Chi interacting with C3b structures (PDB IDs: 7ZGK, 7ZGK, and 3G6J, respectively). The ionic bonds are highlighted in cyan lines and the hydrogen bonds are in hot pink dashed lines.

These results align with our observations on complement activation, where Fuc-NPs demonstrated the capability to activate the complement system. The adsorption of Fuc-NPs to C3b may favor the formation of aggregates and thus reduce the concentration of C3b, which may justify the results presented by Fuc-NPs-RREP with a low concentration of C3 and C3b in the experiment when in high concentrations of nanoparticles (Figure 8). It is believed that C3b interacts not only with the sulfate groups of Fuc; moreover, Fuc-NPs-RREP appear to have fewer sulfate groups on their surface compared to Fuc-NPs-AEP. This reduced number of sulfates may favor a stronger binding of C3b to the surface of Fuc-NPs-RREP, thereby promoting aggregate formation.

In Figure 12C, the Lev structure shows a stable interaction through hydrogen bonds with Lys1051, Gln1055, Glu197, Lys1001, Asp1435, Val1005, and Asp1404. On the other side, Figure 12D shows that Lev interacts with C3b through salt bridges with Glu340 and Lys1478; the poses also show hydrogen bonds with Leu835, Arg834, Ser336, Asp337, Glu1486, Gln340, Glu223, Leu605, and Lys607. All these interactions reinforce the affinity with both, C3 and C3b structures. These results are consistent with those presented in the activation of the complement system, in which the Lev-NPs activated the complement system and that Lev-NPs-RREP, as well as Fuc-NPs-RREP appear to form aggregates with C3b, thereby reducing the concentration of this protein in the experiment.

Conversely, Figure 12E suggests the interaction of Chi with the C3 structure through hydrogen bonds with Lys940, Asn939, Gly1263, Val1005, Ile1004, Lys1436, and Asp1435. On the other hand, Figure 12F exposes the binding of the Chi and C3b structures. The interaction occurs due to a salt bridge on Try815 and hydrogen bonds with Glu320, Gln318, Leu583, Asp315, Ser314, and Glu824. These findings strengthen that they can interact with both C3 and C3b proteins. Although Chi interacts with the C3 and C3b proteins, in the experimental studies on the activation of the complement system, there is a reduced activation compared to other nanoparticles. The presence of amine groups on the surface of Chi-NPs-RREP do not promote strong stabilizing interactions under docking conditions (Figure 12E,F). Therefore, the absence of strong stabilizing contacts involving chitosan amine groups in docking models should be interpreted as a lack of dominant attractive contributions rather than evidence of intrinsic repulsion.

In summary, the molecular docking suggests how the polysaccharides interact with C3 and C3b structures, revealing the importance of polar groups to interact with the residues from the C3 and C3b proteins and indicating the significance of Lys on the binding site to stabilize the interaction through salt bridges. However, it does not account for dynamic rearrangements, protein corona formation, multivalent surface effects, or ionic screening present in serum. Consequently, docking results were interpreted in conjunction with experimental complement activation assays rather than as standalone predictors.

## 4 Conclusions

PIBCA nanoparticles coated with Chi, Fuc, or Lev having positive, negative, and neutral surface charges, respectively, were produced using the AEP and RREP techniques, conferring different architectures of the polysaccharides in their surfaces. As for the adsorption of proteins on the surface of the nanoparticles, the Chi-NPs seem to have a greater interaction with both HSA and serum proteins, indicating the formation of a protein corona with changes in the surface charge of these nanoparticles as well as the Lev-NPs-RREP.

These protein adsorption results complement the complement system activation results, showing that Fuc-NPs and Lev-NPs strongly activated the complement system, with Fuc-NPs-AEP, Fuc-NPs-RREP, and Lev-NPs-AEP standing out. Meanwhile, Lev-NPs-RREP showed weaker activation and no activation of the complement system for Chi-NPs.

Discrepancies observed in the surface conformation of the nanoparticles led to variations in their zeta potential, suggesting a diversity in the chemical groups of the polysaccharides coating the nanoparticle surfaces. These changes induce different profiles of complement activation, in which the conformations of Lev-NPs-AEP and Fuc-NPs-AEP seem to favor complement activation, while Lev-NPs-RREP and Fuc-NPs-RREP seem to induce the formation of aggregates with C3b. The presence of functional groups that interact directly with the C3 and C3b proteins may be a determining factor for the activation of the complement system. According to molecular docking, Fuc, Chi, and Lev interact with C3 and C3b proteins. Fuc interacts with both proteins but interacts preferentially with C3, through sulfate groups with a predominance of ionic bonds. Lev and Chi interact through hydrogen bonds with C3 and C3b proteins.

Taken together, It is believed that the formation of the protein corona may favor a reduction in the activation of the complement system, perhaps by masking chemical groups that contribute to the interaction of these polysaccharides with opsonins, thereby preventing the cleavage of the C3 protein. In this study, it is noteworthy that the load does not seem to be a predominant factor in the activation of the complement system but rather the presence of chemical groups that will interact with plasma proteins, favoring or inhibiting the activation of the complement system.

## Supplementary Material



## Notes

All proteins (C3b and C3) extracted from PDB RSCB database to perform PCA analysis (Table S1); grid box coordinates used in Glide for molecular docking studies against human C3 and C3b complex (Table S2); final volume obtained after counter-dialysis of the nanoparticles, size, PDI, and surface charge before and after counter-dialysis (Table S3); and DLS measurement graphs for AEP-Fuc-NPs (A), RREP-Fuc-NPs (B), AEP-Lev-NPs (C), RREP-Lev-NPs (D), AEP-Chi-NPs (E), and RREP-Chi-NPs (F) (Figure S1) (PDF)
