# Supplementary material for: Activation of the Complement System: Morphological Differences in the Surface of PIBCA Nanoparticles Coated with Polysaccharides
Source: ACS Nanosci Au. 2026 Mar 27;6(3):457–68. doi: 10.1021/acsnanoscienceau.5c00196 (PMC13281202; doi:10.1021/acsnanoscienceau.5c00196)
Supplement: Supplementary file 1 [file ng5c00196_si_001.pdf]

Activation of the complement system: Morphological differences on the surface of PIBCA nanoparticles coated with polysaccharides

Iago Dillion Lima Cavalcanti[1], Vinícius Alexandre Fiaia Costa[2], Danilo Rosa Seixas[3], Bruno Junior Neves[4], Francisco Humberto Xavier-Junior[5], Mariane Cajubá de Britto Lira Nogueira[6*], Nereide Stela Santos Magalhães[7*], Eloisa Berbel Manaia[8*], Gilles Ponchel[9*]

[1]Instituto Keizo-Asami (iLIKA), Universidade Federal de Pernambuco (UFPE), Recife, Pernambuco, postal code: 50670-901, Brazil; Laboratório de Nanotecnologia, Biotecnologia e Cultura de Células (NanoBioCel), Centro Acadêmico de Vitória, Universidade Federal de Pernambuco (CAV/UFPE), Vitória de Santo Antão, Pernambuco, postal code: 55608-000, Brazil; Faculté de Pharmacie Institut Galien Paris-Saclay - UMR-CNRS 8612, Université Paris-Saclay, Orsay, postal code: 91400, France.

[2]Laboratory of Cheminformatics (LabChem), Faculdade de Farmácia, Universidade Federal de Goiás (UFG), Goiânia, Goiás, postal code: 74605-170, Brazil.

[3]Laboratory of Cheminformatics (LabChem), Faculdade de Farmácia, Universidade Federal de Goiás (UFG), Goiânia, Goiás, postal code: 74605-170, Brazil.

[4]Laboratory of Cheminformatics (LabChem), Faculdade de Farmácia, Universidade Federal de Goiás (UFG), Goiânia, Goiás, postal code: 74605-170, Brazil.

[5]Laboratório de Biotecnologia Farmacêutica (BioTecFarm), Departamento de Farmácia, Universidade Federal da Paraíba (UFPB), João Pessoa, Paraíba, postal code: 58051-900, Brazil.

[6]Instituto Keizo-Asami (iLIKA), Universidade Federal de Pernambuco (UFPE), Recife, Pernambuco, postal code: 50670-901, Brazil; Laboratório de Nanotecnologia, Biotecnologia e Cultura de Células (NanoBioCel), Centro Acadêmico de Vitória, Universidade Federal de Pernambuco (CAV/UFPE), Vitória de Santo Antão, Pernambuco, postal code: 55608-000, Brazil.

[7]Instituto Keizo-Asami (iLIKA), Universidade Federal de Pernambuco (UFPE), Recife, Pernambuco, postal code: 50670-901, Brazil.

[8]Faculté de Pharmacie Institut Galien Paris-Saclay - UMR-CNRS 8612, Université Paris-Saclay, Orsay, postal code: 91400, France.

[9]Faculté de Pharmacie Institut Galien Paris-Saclay - UMR-CNRS 8612, Université Paris-Saclay, Orsay, postal code: 91400, France.

Table S1. All proteins (C3b &C3) extracted from PDB RSCB database to perform PCA analysis.

PDB ID	RESOLUTION
7BAG	2 Å
5FO8	2.4 Å
6S0B	2.3 Å
5FOB	2.6 Å
6EHG	2.6 Å
5FO7	2.8 Å
7QIV	2.8 Å
3G6J	3.1 Å
8ENU	3.2 Å
2A73	3.3 Å
5FO9	3.3 Å
7TV9	3.4 Å
8EOK	3.5 Å
7ZGJ	3.6 Å
7ZGK	3.6 Å
2WIN	3.9 Å
6RU5	3.9 Å

Table S2. Grid box coordinates used in Glide for molecular docking studies against human C3 and C3b complex.

C3 & C3b complex PDBs IDs	Center grid box (*x*, *y* and *z* coordinates)
**7ZGK**	170, 170, 140
**3G6J**	25, 78, 27
**5FO8**	30, 77, 30
**6RU5**	-45, 18, 49
**7ZGJ**	170, 175, 140

Table S3. Final volume obtained after counter-dialysis of the nanoparticles, size, PDI, and surface charge before and after counter-dialysis.

**Method**	**Formulation**	**Volume counter-dialysis (mL)**	**Nanoparticles concentration (mg.mL^{-1})**	**Size (nm)**	**PDI**	**Zeta potential (mV)**	**Volume counter-dialysis (mL)**	**Nanoparticles concentration (mg.mL^{-1})**	**Size (nm)**	**PDI**	**Zeta potential (mV)**
		Before counter-dialysis					After counter-dialysis				
AEP	Fuc-NPs	5	7.8 ± 0.2	349.2 ± 5.8	0.05 ± 0.04	-43.1 ± 1.2	~1	77.0 ± 5.3	333.6 ± 2.9	0.05 ± 0.04	-42.7 ± 1.5
	Chi-NPs	4	2.5 ± 1.8	99.0 ± 0.5	0.16 ± 0.01	+52.7 ± 1.3	~2	22.6 ± 0.9	102.0 ± 0.1	0.19 ± 0.03	+43.6 ± 1.1
	Lev-NPs	2	10.0 ± 0.9	432.2 ± 11.6	0.33 ± 0.08	-0.3 ± 0.3	~1	24.6 ± 2.7	422.0 ± 16.1	0.33 ± 0.03	-0.7 ± 0.1
RREP	Fuc-NPs	*ND	30.5 ± 3.6	150.9 ± 2.5	0.20 ± 0.03	-25.8 ± 1.6	*ND	*ND	*ND	*ND	*ND
	Chi-NPs	4	27.4 ± 3.8	296.6 ± 1.1	0.05 ± 0.02	+57.1 ± 2.7	~2	65.1 ± 3.3	349.5 ± 5.6	0.06 ± 0.04	+51.9 ± 1.1
	Lev-NPs	2	12.7 ± 3.3	659.9 ± 39.0	0.07 ± 0.05	-5.2 ± 0.4	~1	29.0 ± 2.0	647.0 ± 33.3	0.05 ± 0.04	-9.7 ± 0.6

*ND= Non-determined; AEP= Anionic Emulsion Polymerization; RREP= Redox Radical Emulsion Polymerization

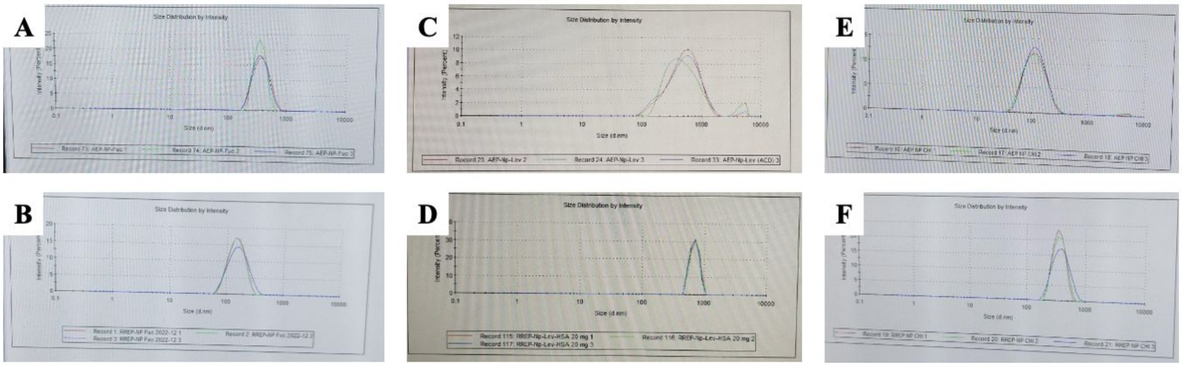

Figure S1. DLS measurement graphs for AEP-Fuc-NPs (A), RREP-Fuc-NPs (B), AEP-Lev-NPs (C), RREP-Lev-NPs (D), AEP-Chi-NPs (E), and RREP-Chi-NPs (F).
